# Synergistic effects of peripheral GABA and GABA-transaminase inhibitory drugs on food intake control and weight loss in high-fat diet-induced obese mice

**DOI:** 10.3389/fphar.2024.1487585

**Published:** 2024-10-02

**Authors:** Tomoka Nagao, Jason D. Braga, Siyi Chen, Masubon Thongngam, Maesaya Chartkul, Noriyuki Yanaka, Thanutchaporn Kumrungsee

**Affiliations:** ^1^ Program of Food and AgriLife Science, Graduate School of Integrated Sciences for Life, Hiroshima University, Hiroshima, Japan; ^2^ Institute of Food Science and Technology, College of Agriculture, Food, Environment and Natural Resources, Cavite State University, Indang, Philippines; ^3^ Department of Food Science and Technology, Faculty of Agro-Industry, Kasetsart University, Bangkok, Thailand; ^4^ Weight Care Clinic, Health Promotion Center, Bangkok Chanthaburi Hospital, Chanthaburi, Thailand; ^5^ Smart Agriculture, Graduate School of Innovation and Practice for Smart Society, Hiroshima University, Hiroshima, Japan

**Keywords:** GABA, GABA-T, food intake, obesity, weight loss, semaglutide, appetite, vigabatrin

## Abstract

**Background:**

Developing anti-obesity interventions targeting appetite or food intake, the primary driver of obesity, remains challenging. Here, we demonstrated that dietary γ-aminobutyric acid (GABA) with GABA-degradation inhibitory drugs could be an anti-obesity intervention possessing strong food intake-suppressive and weight-loss effects.

**Methods:**

High-fat (HF)-diet-induced obese mice were divided into six groups receiving either the HF diet or the 2% GABA-HF diet with daily administration of PBS or the GABA-degradation inhibitory drugs, vigabatrin and ethanolamine-O-sulfate (EOS). In 24-h fast-induced refeeding, lean mice with a basal diet were used, and food intake was measured from 0.5 to 24 h after refeeding.

**Results:**

Coadministration of the 2% GABA-HF diet with vigabatrin or EOS significantly decreased food intake (−53%, −35%) and body weight (−22%, −16%) within 11 days in obese mice, along with a marked increase in plasma GABA levels. Mice receiving dietary GABA alone or the drugs alone exhibited no such effects. Hypothalamic GABA levels increased in drug-treated mice, regardless of diet. At 0.5 h after refeeding, food intake was similar in all groups. However, at 0.5 h, plasma GABA levels were markedly increased only in mice receiving coadministration of dietary GABA and the drugs, and their food intake was completely inhibited for over 6 h, while mice in other groups gradually increased their food intake.

**Conclusion:**

Combining dietary GABA with GABA-degradation inhibitory drugs effectively suppresses food intake and promotes weight loss in obese mice, primarily through increased plasma GABA availability. These findings may advance the development of food intake-controlling strategies for obesity management.

## 1 Introduction

Overeating and imbalanced energy expenditure are major factors contributing to overweight and obesity. Theoretically, reducing food intake and increasing energy expenditure are the simplest methods for treating obesity. However, controlling food intake for weight loss is often challenging to achieve and maintain for obese individuals. Currently, development of anti-obesity drugs or interventions that suppress appetite or reduce food intake—the direct and primary causes of obesity—remains challenging. In 2021, semaglutide was approved as a new and effective weight-loss drug, which exerts its anti-obesity effects by strongly reducing appetite and suppressing food intake (Wilding et al., 2021; Shu et al., 2022). Despite its strong efficacy, incomplete understanding of its mechanisms, along with concerns over safety and high cost, likely limit its widespread use. Therefore, the development of new appetite suppressant drugs and interventions is still necessary.

The human body regulates food intake and appetite in a highly complex manner through communication between the gut (peripheral controls) and the brain (central controls) (Hussain et al., 2014). Peripheral signals convey information from the gut to the brain via two main routes: the bloodstream and the vagus nerve. Peripheral signals, such as nutrients and hormones, travel through the bloodstream and, upon reaching the brain, act on the hypothalamus—specifically the arcuate nucleus (ARC), where the blood-brain barrier is incomplete (Hussain et al., 2014). The hypothalamic ARC houses two distinct populations of neurons: agouti-related peptide (AgRP)-expressing neurons and pro-opiomelanocortin (POMC)-expressing neurons. These neurons play crucial roles in integrating peripheral and central signals by releasing various neuropeptides (e.g., AgRP, neuropeptide Y (NPY), alpha-melanocyte-stimulating hormone (α-MSH)) and neurotransmitters (e.g., γ-aminobutyric acid (GABA) and glutamic acid (Glu)) to nearby and downstream neurons, both within and outside the ARC, to regulate appetite and food intake in a coordinated manner (Wu and Palmiter, 2011; Vong et al., 2011; Lowell, 2019). Conversely, peripheral signals carrying gut information via the vagus nerve are transmitted to the brainstem. The brainstem then projects these peripheral inputs to the hypothalamus and other brain regions to regulate appetite and food intake. In a bidirectional manner, the hypothalamus also sends information back to the brainstem, which in turn relays this information through the vagus nerve back to the gut to control, such as, gastric emptying, gastric motility, and pancreatic secretions. To develop anti-obesity drugs or interventions, targeting or manipulating the signals of these neuropeptides or neurotransmitters—either by enhancing or inhibiting them—can be an effective strategy for controlling food intake.

Research suggests that central GABAergic signaling plays a complex role in regulating food intake and energy homeostasis. Depending on the brain region and neuron type, GABA can either suppress or promote food intake and energy expenditure. For example, GABA signaling from hypothalamic AgRP neurons projecting to the dorsomedial hypothalamic nucleus, paraventricular nucleus of the hypothalamus, and parabrachial nucleus promotes feeding (Han et al., 2023; Lowell, 2019; Wu et al., 2009). Deletion of GABA synthesis and vascular transporters in hypothalamic AgRP neurons has been shown to reduce food intake and increase energy expenditure (Meng et al., 2016; Tong et al., 2008). Conversely, GABAergic outputs from AgRP neurons to the dorsal bed nucleus of the stria terminalis were found to reduce food intake and body weight (Xia et al., 2021), while activation of GABAergic neurons in the lateral hypothalamus increased body temperature (de Vrind et al., 2019). Enhanced GABA signaling in the nucleus accumbens has been proposed to reduce the dopamine reward response associated with various addictions, including food addiction (Kumrungsee, 2024). In peripheral system, GABA was found to improve glucose metabolism and functions of hormones involved in energy expenditure and food intake regulation, such as thyroid hormones and insulin (Xie et al., 2014; Tian et al., 2011; Soltani et al., 2011; Jin et al., 2024). GABA also suppresses food intake by sending peripheral signals to the brainstem via the vagus nerve (Nakamura et al., 2022). Additionally, GABA was shown to increase lipolysis and white adipose tissue browning, while decreasing adipogenesis and lipogenesis (Jin et al., 2024).

In our previous study, we accidentally found that a high dose (5%) of GABA mixed into a basal diet significantly decreased food intake (−30%) and body weight (−25%) in lean mice, along with an elevation in blood GABA levels, within 6 weeks. In contrast, a lower dose of GABA (2%) had no effect on food intake, body weight, or blood GABA levels (Kumrungsee et al., 2020; Sato et al., 2021). We hypothesized that the food intake-suppressive effect might be attributed to elevated blood GABA levels. To test this hypothesis, lean mice were fed the 2% GABA diet, which had previously shown no effects on food intake and blood GABA levels, and administered vigabatrin—an anti-epileptic drug that inhibits GABA-transaminase, a GABA-degrading enzyme highly expressed in the brain and liver. Notably, coadministration of dietary GABA and the GABA-T inhibitor vigabatrin strongly suppressed food intake (−50%) and body weight (−28%), while causing a remarkable elevation in blood GABA levels within 2 weeks. These effects were absent in mice fed with the GABA diet alone, without vigabatrin administration (Sato et al., 2021). These findings suggest that increased blood GABA availability may have a promising anti-obesity action by strongly suppressing food intake, similar to the action of the new anti-obesity drug semaglutide.

However, it is necessary to address the following points: whether this strong food intake-suppressive effect is solely attributed to vigabatrin alone (without peripheral GABA involvement), whether other GABA-T inhibitors can produce effects similar to those of vigabatrin, and, importantly, whether coadministration of dietary GABA and GABA-T inhibitory drugs can exert anti-obesity effects in obese mice. Therefore, in the present study, we evaluated whether the GABA-T inhibitory drugs, vigabatrin and ethanolamine-O-sulfate (EOS), could produce these effects in high-fat (HF) diet-induced obese mice fed with the 2% GABA-HF diet. Blood GABA and hypothalamic GABA levels were measured to determine the potential sites of action, whether at peripheral and/or central locations. Additionally, plasma glucose and lipid parameters, fat mass, and muscle mass were measured to assess fat utilization.

## 2 Materials and methods

### 2.1 Animals and diets

Male CD1 mice (8 weeks old) were purchased from Charles River Japan (Hino, Japan) and acclimatized on a non-purified commercial rodent diet (MF, Oriental Yeast Co., Ltd., Tokyo, Japan) for 1 week. After acclimatization, all mice were switched to a high-fat diet (HFD-60, 60% of calories from fat, Oriental Yeast) for 9 weeks to induce obesity. During this period, the mice were housed in metal cages, with two mice per cage. At the end of the 9-week HF diet feeding, 6-h fasted blood glucose levels were measured using an Accu-Chek (Roche Diabetes Care) and compared with those of non-HF-diet-fed mice. The HF-diet-fed mice were then subjected to a food intake study as described below.

For the fast-induced refeeding study, male CD1 mice (8 weeks old) were purchased from Charles River Japan and acclimatized on the MF diet for 1 week. The mice were then housed in metal cages (two mice per cage) and fed a basal diet for 1 week. The basal diet consisted of the following components (g/kg diet): α-cornstarch, 402; casein, 200; sucrose, 200; corn oil, 100; cellulose, 50; AIN-93G mineral mixture, 35; AIN-93 vitamin mixture, 10; and L-cystine, 3, as previously described (Sato et al., 2021). After this period, the mice were subjected to a fast-induced refeeding study detailed below.

All the mice used in this study were maintained in accordance with the Guide for the Care and Use of Laboratory Animals established by Hiroshima University, and the procedures were approved by the Ethics Committee of the University (Ethical Approval No. C22-31-2). Mice were housed in a temperature-controlled room (24°C ± 1°C) with a 12 h light/dark cycle (lights on from 08:00–20:00) with free access to food and drinking water.

### 2.2 A food intake study

After 9 weeks of HF diet feeding, all mice were divided into six groups and received either the HF diet or the 2% GABA-HF diet (Oriental Yeast Co., Ltd.), along with daily administration of PBS (Nacalai Tesque, Kyoto, Japan), vigabatrin (Sabril^®^, Sanofi Aventis, Patheon, France), or EOS (Sigma-Aldrich, St. Louis, MO). Food intake and body weight were measured daily at 16:00, followed by subcutaneous injections of PBS, vigabatrin (250 mg/kg), or EOS (250 mg/kg) at 17:00, with the diets provided at 18:00. To monitor food intake during the dark and light cycles, measurements were taken at 18:00, 21:00, 24:00, 8:00, 12:00, and 16:00 for three random days. On day 11 of treatment, when the body weight of mice receiving the 2% GABA-HF diet with the drugs decreased by 20%, the experiment was terminated for animal welfare considerations, and tissue samples were collected. All mice were fasted for 6 h before being sacrificed under isoflurane anesthesia (between 13:00 and 16:00). No drugs were administered on the day of sacrifice, with the last injection occurring at 17:00 on day 10. Blood samples were collected from the tails between 8:00 and 10:00 and from abdominal veins at the time of sacrifice between 13:00 and 16:00. Plasma was obtained by centrifugation at 3,000 × g for 10 min and stored at −80°C until analysis. Brain tissues, fat tissues, liver, and skeletal muscle tissues were immediately harvested, weighed, snap-frozen in liquid nitrogen, and stored at −80°C until analysis.

### 2.3 Plasma glucose and lipid analysis

Levels of glucose, triacylglycerols, free fatty acids, ketone bodies, and total cholesterol were determined using a Beckman Coulter AU480 analyzer (Beckman Coulter, Krefeld, Germany), an automated chemistry instrument for turbidimetric, spectrophotometric, and ion-selective electrode measurements (Sato et al., 2021). Briefly, 200 μL of plasma was used to measure these parameters according to the manufacturer’s protocol.

### 2.4 Plasma and brain GABA analysis

Brain tissues (hypothalamus, hippocampus, and cortex) were homogenized in methanol with an internal standard (20 μM methionine sulfone (MS)) as previously reported (Kumrungsee et al., 2019; Soga et al., 2003). Then, supernatants were evaporated to dryness and resuspended in methanol prior to analysis in UPLC-MS/MS (Waters, Milford, MA). For plasma samples, plasma was mixed with 20 μM-MS containing methanol at a ratio of 1:3 of plasma to methanol, followed by centrifugation, filtration, and injection to UPLC-MS/MS. Liquid chromatography was performed at 30°C using an Acquity UPLC BEH C18 (1.7 μm, 2.1 × 50 mm) column (Waters) and a gradient system consisting of mobile phase A (5 mM perfluoroheptanoic acid (PFHpA; Sigma-Aldrich, Louis, MO)) in MilliQ water and mobile phase B (5 mM PFHpA in methanol) at a flow rate of 400 μL/min. The gradient program was set at the following conditions: 0–0.5 min, 5%–40% B; 0.5–10.5 min, 4050% B; 10.5–11 min, 50%–100% B; 11–12 min, 100% B; 12–12.5 min, 100%–5% B; and 12.5–17.5 min, 5% B. Total run-to-run time was 17.5 min with an injection volume of 5 μL. Mass spectrometric analysis was performed by multiple reaction monitoring (MRM) in the ESI-positive mode, 400°C desolvation temperature, 120°C source temperature, nitrogen as desolvation and cone gas, and argon as the collision gas for MRM and daughter-ion scans. The details of the capillary voltage, cone voltage, and MRM for GABA (Nacalai Tesque) were set as previously reported (Kumrungsee et al., 2020).

### 2.5 GABA-T activity analysis

Liver GABA-T activity was examined following previously established protocols (Kumrungsee et al., 2020; De Ita-Pérez et al., 2014; Blancquaert et al., 2016) with some modifications. Briefly, liver tissue was homogenized in 10 volumes of 50 mM phosphate buffer (pH 7.0) at 13,000 rpm for 30 s on ice, followed by centrifugation at 16,000 g for 15 min at 4°C to collect the supernatant. Subsequently, 10 μL of supernatant was incubated with 190 μL of 100 mM phosphate buffer (pH 8.6) containing 1 mM NAD+ (Nacalai Tesque), 5 mM α-ketoglutarate (Nacalai Tesque), 3.5 mM mercaptoethanol (Nacalai Tesque), and 6 mM GABA (Nacalai Tesque) for 45 min at 30°C. The same mixture without GABA was run in parallel as a blank. The reaction was then terminated by heating at 85°C for 5 min, followed by centrifugation at 10,000 rpm for 1 min. The supernatant was used for NADH measurement. Since NAD+ is reduced to NADH during the reaction, the enzymatic reaction rate was determined by measuring NADH production using a spectrophotometer (Jasco, Tokyo, Japan) at 340 nm.

### 2.6 A fast-induced refeeding study

After acclimatization with the basal diet for 1 week, all mice were divided into six groups and received either the basal diet or the 2% GABA-basal diet with PBS, vigabatrin, or EOS administration. The diets and the drugs were administrated daily, similar to aforementioned in the food intake study above. On day 3 after the treatment, when decreases in food intake and body weight were constantly confirmed in mice receiving the 2% GABA diet with the drugs, at 10:00 in the morning, food were removed to fast those mice (without drug administration). Then, on day 4, at 9:00, PBS, vigabatrin (250 mg/kg), or EOS (250 mg/kg) was subcutaneously injected to mice, followed by refeeding at 10:00. Food intake was measured at 0.5, 1, 2, 3, 6, 9, 12, and 24 h after refeeding. Then, all mice were washed out by giving the MF diet for 2 weeks. To determine blood GABA levels at each time point after refeeding, the fast-induced refeeding experiment was conducted again. Then, blood was collected from tails at 0, 0.5, 6, 12, and 24 h after refeeding. Plasma preparation and plasma GABA levels were then measured as the methods mentioned above.

### 2.7 Statistical analysis

All values are expressed as the means ± SEM. A two-way ANOVA followed by Tukey’s multiple comparisons test was used to test significances between groups. Pearson correlation analysis (two-tailed) was performed to determine the correlative relationship. All statistical analyses were done by using GraphPad Prism 10 (GraphPad Software, CA, United States). *p* < 0.05 was considered statistically significant.

## 3 Results

### 3.1 Dietary GABA and GABA-T inhibitory drugs synergistically suppress food intake and induce body weight loss

**FIGURE 1:**
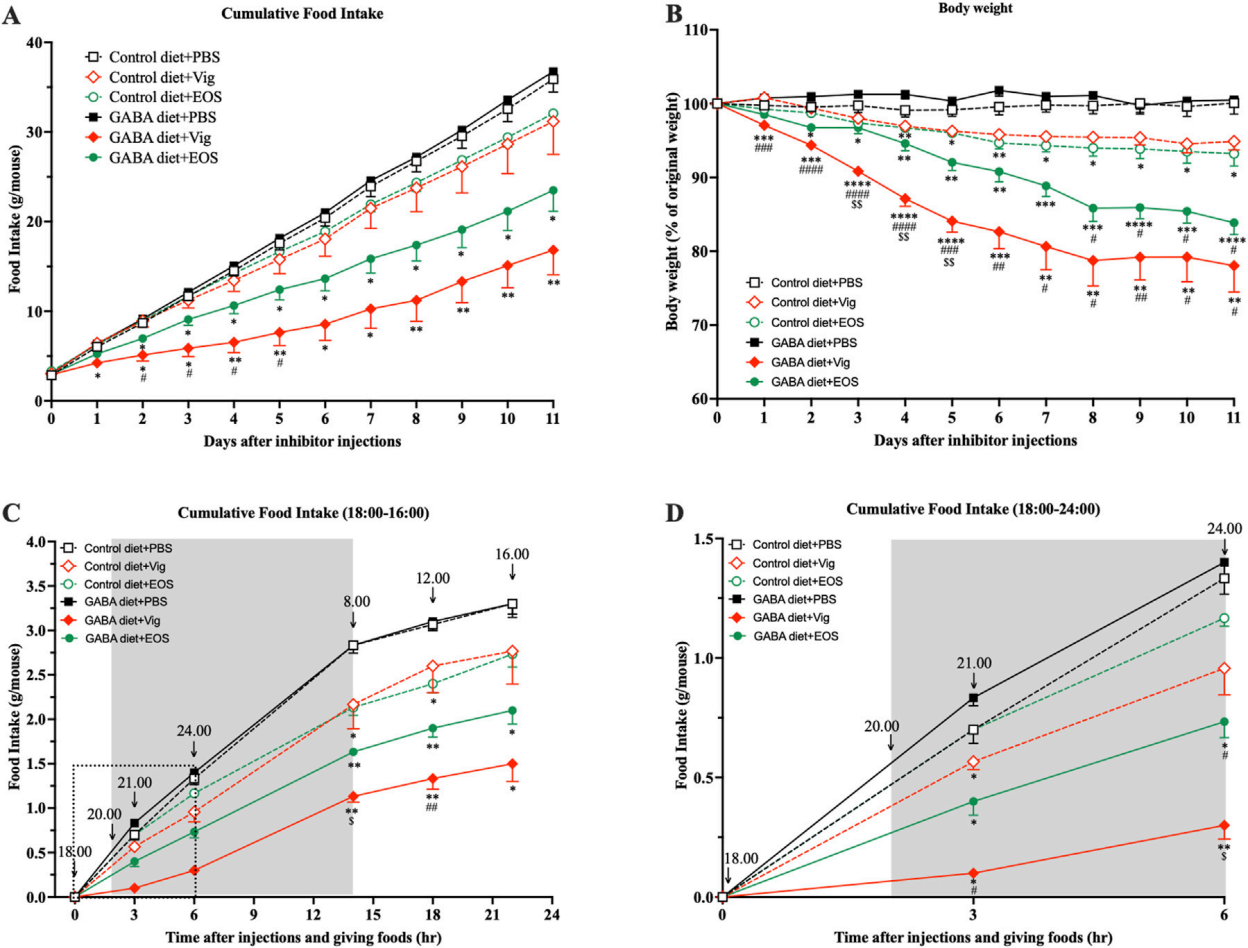
Coadministration of dietary GABA and GABA-T inhibitory drugs suppresses food intake and induces body weight loss. **(A)** Cumulative food intake and **(B)** percentage of initial body weight during 11 days of PBS or the GABA-T inhibitory drug (vigabatrin or EOS) injections (250 mg/kg s.c. daily) in obese mice receiving a high-fat diet or a high-fat plus 2%GABA diet. **(C)** Cumulative food intake assessed at 21:00, 24:00, 8:00, 12:00, and 16:00 after daily food and drug administrations at 18:00. Data are the average of three random days. **(D)** The zoomed version of **(C)** presenting cumulative food intake assessed at 21:00 and 24:00. All data were analyzed using a two-way ANOVA, followed by Tukey’s multiple comparisons test. ^*^ *p* < 0.05, ^**^ *p* < 0.01, ^***^ *p* < 0.001, ^****^ *p* < 0.0001 *versus* Control diet + PBS and GABA diets + PBS groups. ^#^ *p* < 0.05, ^##^ *p* < 0.01, ^###^ *p* < 0.001, ^####^ *p* < 0.0001 *versus* Control diet + Vig and Control diet + EOS groups. ^$^ *p* < 0.05, ^$$^ *p* < 0.01 *versus* a GABA diet + EOS group. Results are presented as mean ± SEM values (n = 8–9).

Obese mice induced by 9 weeks of HF diet feeding were used in this study. At the end of the HF diet feeding, the blood glucose levels of HF-diet-fed mice increased to 144.7 mg/dL, compared to 112.4 mg/dL in non-HF-diet-fed mice (data not shown). This increase in blood glucose in obese mice was consistent with the levels reported by the HF diet manufacturer (Oriental Yeast Co., Ltd.). These obese mice were then divided into six groups, receiving either an HF diet (control) or an HF plus 2% GABA diet with or without vigabatrin or EOS administration. We observed that daily administration of the GABA-T inhibitory drug vigabatrin or EOS to the mice fed with the 2% GABA-HF diet (GABA diet + Vig and GABA diet + EOS) resulted in a significant, progressive decline in food intake by 53% (vigabatrin) and 35% (EOS), leading to a substantial reduction in body weight by 22% (vigabatrin) and 16% (EOS) within 11 days, compared to the HF diet (Control diet + PBS) (Figures 1A,B). In contrast, these food-intake suppressive and weight-loss effects were completely abolished in mice that received only the 2% GABA-HF diet (GABA diet + PBS) without vigabatrin or EOS administration (Figures 1A,B). Notably, without dietary GABA, the drug vigabatrin or EOS alone (Control diet + Vig and Control diet + EOS) exhibited only slight effects on food intake (−13% for vigabatrin and −11% for EOS) and body weight (−5% for vigabatrin and −7% for EOS) (Figures 1A,B). This suggests that the strong food intake-suppressive and weight-loss effects are not solely attributed to either vigabatrin or EOS alone, nor to dietary GABA alone. Instead, these effects are likely due to the synergistic action of dietary GABA and the drug vigabatrin or EOS.

Next, we investigated whether coadministration of dietary GABA and the GABA-T inhibitory drugs suppresses food intake during the dark or light cycle. As shown in Figures 1C,D, coadministration of dietary GABA with either vigabatrin or EOS led to a reduction in food consumption during the initial 3-h (21:00) and 6-h (24:00) periods following drug administration and feeding at 18:00, compared to the HF control diet group. This food intake-suppressive effect continued until the end of the dark cycle at 8:00. Vigabatrin or EOS alone had a slight suppressive effect during the 18:00-8:00 period. During the light cycle (8:00-16:00), all groups of mice consumed a similarly small amount of food (∼0.5 g/mouse). These results suggest that the coadministration of dietary GABA with vigabatrin or EOS primarily suppresses food intake during the dark cycle, with this suppressive action persisting into the light cycle, as indicated by the lack of compensatory feeding during the light cycle.

### 3.2 Dietary GABA and GABA-T inhibitory drugs synergistically elevate plasma GABA levels without further increasing brain GABA levels

**FIGURE 2:**
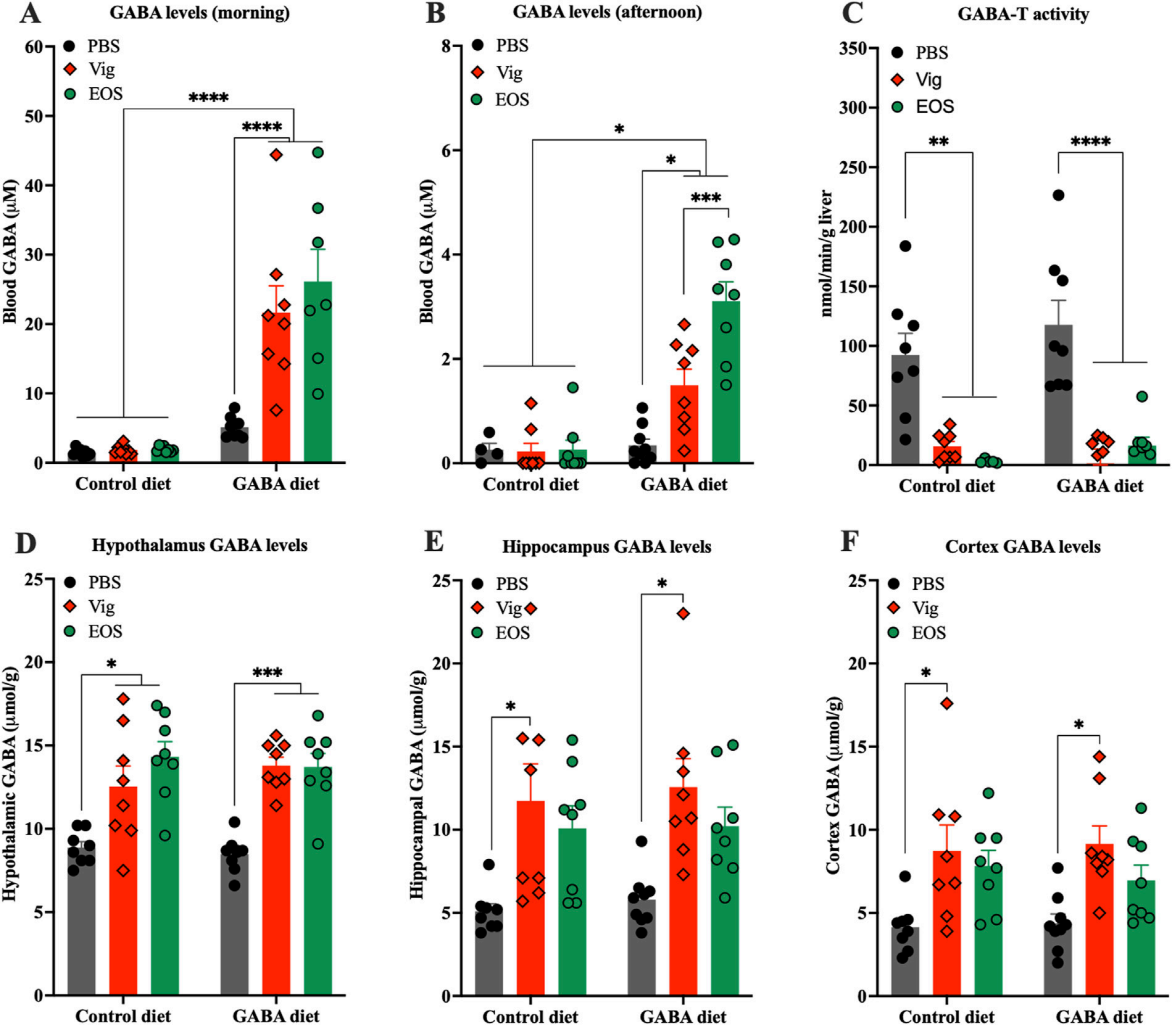
Effects of coadministration of dietary GABA and GABA-T inhibitory drugs on plasma and hypothalamic GABA levels and hepatic GABA-T activity. GABA levels in plasma collected from **(A)** tails between 8:00 and 10:00 and from **(B)** abdominal veins at sacrifice between 13:00 and 16:00. **(C)** GABA-T activity in liver tissues. GABA levels in hypothalamus **(D)**, hippocampus **(E)**, and cortex **(F)**. All data were analyzed using a two -way ANOVA, followed by Tukey’s multiple comparisons test. ^*^ *p* < 0.05, ^***^ *p* < 0.001, ^****^ *p* < 0.0001. Results are presented as mean ± SEM values (n = 8–9).

To determine the preliminary potential sites of action, whether in the peripheral system alone or also in the central system, we measured GABA levels in plasma and hypothalamus, the gate keeper in the control of food intake and appetite (Hussain et al., 2014). At the end of the dark cycle (8:00-10:00), significantly elevated plasma GABA levels were observed only in mice that received coadministration of dietary GABA and the drug (21.6 ± 3.9 µM for vigabatrin and 26.1 ± 4.6 µM for EOS, compared to 1.5 ± 0.2 µM for the HF diet alone and 5.3 ± 0.4 µM for the 2% GABA-HF diet alone) (Figure 2A). In contrast, dietary GABA alone or the drug alone had little or no effects on increasing plasma GABA levels (Figure 2A). These elevated plasma GABA levels resulting from coadministration of dietary GABA and the drug persisted until the afternoon (Figure 2B), albeit with an approximate 10-fold decrease compared to the levels observed in the morning. A decrease in hepatic GABA-T activity due to the administration of vigabatrin or EOS was also confirmed (Figure 2C).

In the brain, both vigabatrin and EOS administrations significantly increased GABA levels in the hypothalamus, regardless of whether the mice were on the HF control diet or the GABA-HF diet (Figure 2D). This result suggests that the GABA-T inhibitory drugs (vigabatrin and EOS) penetrated the brain, inhibited GABA-T activity, and thereby prevented brain GABA degradation, leading to an increase in brain GABA levels. These increased GABA levels were not only found in the hypothalamus but also in other brain regions, such as the cortex and hippocampus (Figures 2E,F).

### 3.3 Dietary GABA and GABA-T inhibitory drugs synergistically enhance fat utilization

As shown in Table 1, coadministration of dietary GABA with the drug vigabatrin or EOS significantly decreased white adipose tissue weights without affecting skeletal muscle weights or liver weights, compared to all other groups. Then, we further examined plasma lipid and glucose profiles and found that coadministration of dietary GABA with the drug vigabatrin or EOS significantly decreased plasma levels of free fatty acids, ketone bodies, and glucose, while triacylglycerol and total cholesterol levels remained unchanged across the groups. The lower plasma levels of fatty acids and ketone bodies in these mice might indicate increased beta-oxidation or heightened fat utilization.

**TABLE 1:** Body and tissue weights and plasma glucose and lipid profiles.

	Control diet +PBS	Control diet +Vig	Control diet +EOS	GABA diet +PBS	GABA diet +Vig	GABA diet +EOS
Body weights and tissue weights						
Initial body weight (g)	45.8 ± 1.0	43.7 ± 1.3	45.0 ± 2.1	45.6 ± 2.1	44.7 ± 0.9	44.5 ± 2.0
Final body weight (g)	45.9 ± 1.3^a^	41.4 ± 1.0^abc^	41.8 ± 1.7^ac^	45.7 ± 1.9^a^	34.9 ± 1.8^b^	37.1 ± 1.3^bc^
Epididymal white adipose tissue (mg/g body weight)	47.2 ± 9.0^a^	20.5 ± 3.9^bc^	20.8 ± 4.4^bc^	34.4 ± 4.4^ac^	10.5 ± 2.8^b^	15.8 ± 3.3^bc^
Gastrocnemius muscle (mg/g body weight)	4.1 ± 0.1	4.1 ± 0.2	4.4 ± 0.1	4.3 ± 0.2	4.6 ± 0.3	4.6 ± 0.3
Soleus muscle (mg/g body weight)	0.18 ± 0.01	0.18 ± 0.02	0.21 ± 0.01	0.21 ± 0.03	0.21 ± 0.02	0.17 ± 0.01
Liver (mg/g body weight)	37.8 ± 2.7	33.2 ± 0.9	34.0 ± 1.5	38.4 ± 4.0	31.7 ± 2.7	32.4 ± 2.7
Plasma glucose and lipid profiles						
Triacylglycerols (mg/dL)	28.0 ± 4.4	32.9 ± 4.5	22.8 ± 2.4	24.1 ± 6.4	21.7 ± 4.2	21.5 ± 2.3
Free fatty acids (mEq/L)	1218 ± 65^a^	1093 ± 44^a^	1211 ± 58^a^	1220 ± 59^a^	834 ± 82^b^	1015 ± 53^ab^
Ketone bodies (μmol/L)	605.7 ± 19.1^ac^	555.6 ± 68.7^ac^	576.7 ± 88.9^a^	535.6 ± 86.8^ac^	230.1 ± 58.4^b^	267.0 ± 52.8^bc^
Total cholesterol (mg/dL)	178.1 ± 18.9	142.7 ± 7.0	187.3 ± 6.3	183.5 ± 14.7	153.6 ± 14.4	189.7 ± 7.3
Glucose (mg/dL)	232.6 ± 15.4^ab^	176.7 ± 9.9^ac^	177.2 ± 10.5^ac^	242.5 ± 13.2^b^	154.6 ± 18.1^c^	163.5 ± 16.0^ac^

Results are presented as mean ± SEM, values (n = 8–9). Values without a common superscript letter are significantly different, *p* < 0.05, analyzed by using a two-way ANOVA, followed by Tukey’s multiple comparisons test.

### 3.4 Dietary GABA and GABA-T inhibitory drugs synergistically completely inhibit food intake for over 6 h with elevation of plasma GABA levels after fast-induced refeeding

**FIGURE 3:**
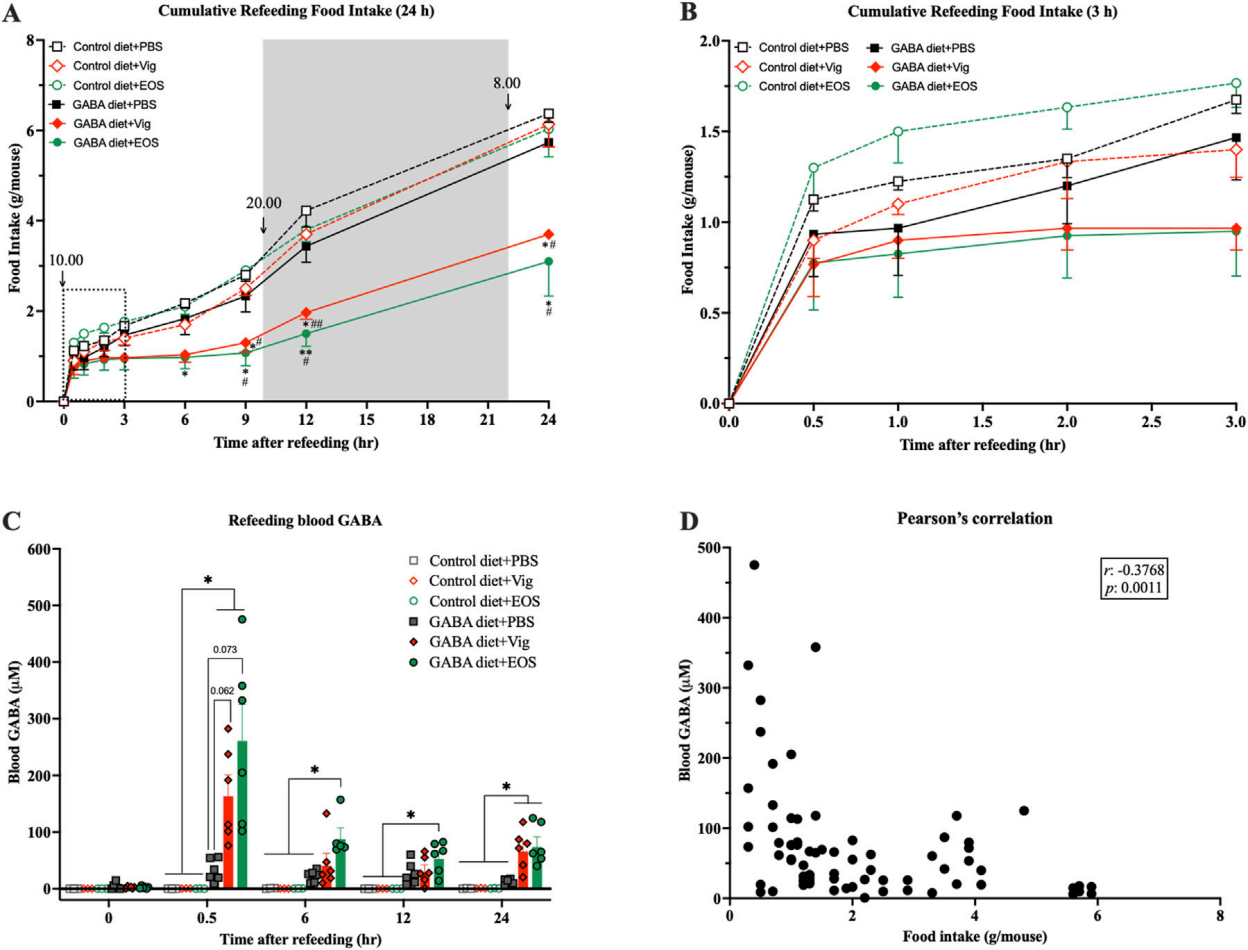
Coadministration of dietary GABA and GABA-T inhibitory drugs suppresses food intake over 6 h after refeeding and increases plasma GABA levels. Mice were fasted for 24 h, and the drugs were administrated 1 h before refeeding. **(A)** Cumulative food intake assessed 0.5, 1, 2, 3, 6, 9, 12, and 24 h after refeeding. **(B)** The zoomed version of **(A)** presenting cumulative food intake assessed 0.5–3 h after refeeding. ^*^ *p* < 0.05, ^**^ *p* < 0.01 *versus* Control diet + PBS and GABA diets + PBS groups. ^#^ *p* < 0.05, ^##^ *p* < 0.01, ^###^ *p* < 0.001 *versus* Control diet + Vig and Control diet + EOS groups. **(C)** Plasma GABA levels at each time point during refeeding. ^**^ *p* < 0.01. **(D)** A correlation between plasma GABA levels and food intake from **(A)** and **(C)**. Data in **(A–C)** were analyzed using a two-way ANOVA, followed by Tukey’s multiple comparisons test. Data in **(D)** were analyzed using Pearson analysis. Results are presented as mean ± SEM values (n = 6).

To further confirm the food intake-suppressive effect and explore the correlation between food intake and plasma GABA levels resulting from coadministration of dietary GABA with the drug vigabatrin or EOS, a 24-h fast-induced refeeding experiment was conducted. We found that after the first 30 min of refeeding, all groups consumed a similar amount of food (∼1 g/mouse) (Figures 3A,B). However, between 30 min and 6 h, mice receiving coadministration of dietary GABA with the drug vigabatrin or EOS barely consumed any food, while mice receiving either the drug alone or dietary GABA alone gradually increased their food intake (∼2 g/mouse at 6 h), similar to the control HF-diet fed mice (Figures 3A,B). At 9 h after refeeding (19:00), just before the beginning of the dark cycle (20:00), food intake increased significantly in all groups, but the increase in mice receiving coadministration of dietary GABA with the drug vigabatrin or EOS remained lower than that of the other groups until 24 h (Figure 3A).

When measuring plasma GABA levels, at 30 min after refeeding, plasma GABA levels were markedly increased in mice receiving coadministration of dietary GABA with the drug vigabatrin or EOS (167.0 ± 33.8 µM for vigabatrin and 264.5 ± 60.6 µM for EOS vs 0.03 ± 0.03 µM for the HF diet alone and 32.2 ± 7.9 µM for the 2% GABA-HF diet alone) (Figure 3C). These elevated plasma GABA levels decreased by 6 h after refeeding (Figure 3C). Correlation analysis showed a significant negative association between plasma GABA levels and food intake (*r* = −0.3768, *p* = 0.0011, Figure 3D). These results suggest that the increased plasma GABA levels in mice receiving coadministration of dietary GABA with the drug vigabatrin or EOS likely induced a chronic food intake-suppressive effect, persisting for at least 6 h after the marked increase in plasma GABA levels.

## 4 Discussion

In the present study, we addressed unresolved questions regarding whether the food intake-suppressive and weight-loss effects are solely attributed to vigabatrin and whether such effects can be observed in obese mice. Our findings suggest the following conclusions. First, the food intake-suppressive and weight-loss effects are not exclusively due to either the GABA-degradation inhibitory drug (vigabatrin or EOS) alone or dietary GABA alone. Instead, these effects result from the synergistic action of dietary GABA and the drugs. Both drugs, vigabatrin and EOS, can exert the similar effects, indicating that these outcomes are not unique to vigabatrin alone. This finding suggests that compounds with GABA-T inhibitory action have the potential to exhibit these effects. Second, our study demonstrates that coadministration of dietary GABA with the drugs can also exert food intake-suppressive and weight-loss effects in obese mice. This observed body weight loss is likely due primarily to a reduction in fat mass rather than a reduction in lean mass, as skeletal muscle weights remained unaffected. Given the decreased plasma levels of free fatty acids, ketone bodies, and glucose observed with the coadministration of dietary GABA and the drugs, it is possible that mice receiving this combination may compensate for the low energy status resulting from reduced food intake by enhancing fat utilization for energy production.

Third, increased blood GABA is likely a key effector underlying the food intake-suppressive and weight-loss effects. Elevated blood GABA levels can strongly suppress food intake for 6 h, with this suppressive effect being sustained over 24 h without evidence of compensatory feeding. Consequently, the overall 24-h food intakes of mice receiving dietary GABA combined with the drugs are significantly lower than those of mice in other groups (Figure 3A). From 9 h (19:00), before the onset of the dark cycle (20:00), to 12 h (22:00) after refeeding, food intake significantly increased in all groups, followed by a gradual rise until 24 h (10:00) (Figure 3A). During the 9–24 h period after refeeding, the rate of food intake (slope of the graph in Figure 3A) in mice receiving dietary GABA with the drugs remained lower or comparable to that of mice in other groups, despite having consumed much less food during the 0.5–6 h period after refeeding. It is plausible that the markedly increased blood GABA levels observed at 0.5 h after refeeding might enhance and prolong satiety, rather than causing an adverse sensation that could lead to compensatory feeding once that adverse sensation diminishes as blood GABA levels decrease. In rodents, vigabatrin is rapidly eliminated from the blood, with concentrations almost completely reduced by 8 h (dropping from 1000 to 10 µM) and an elimination half-life (*t*_1/2_) of 0.8–1.7 h (Tong et al., 2007). Considering our findings, it is possible that by 6 h after refeeding (7 h after vigabatrin administration), vigabatrin might have been nearly eliminated from the blood, leading to a reduction in its GABA-degradation inhibition, a subsequent decrease in plasma GABA levels (Figure 3C), and the start of increased food intake (Figure 3A) at 6 h after refeeding.

Giving that vigabatrin can cross the BBB, inhibiting GABA-T activity and regulating GABA levels or signals in the brain, one might argue that vigabatrin possibly regulates food intake or appetite by acting directly in the brain. However, mice receiving vigabatrin alone (without dietary GABA) had higher food intake than those receiving both vigabatrin and dietary GABA (Figures 1A,D, 3A). Although hypothalamic GABA levels were elevated in both groups treated with vigabatrin (Figure 2D), plasma GABA levels increased only in mice that received both vigabatrin and dietary GABA (Figures 2A,B). Notably, the administration of vigabatrin or EOS did not further increase hypothalamic GABA levels in mice that received dietary GABA (Figure 2D), suggesting that peripheral or plasma GABA has low potency in crossing the blood-brain barrier (BBB). These results imply that elevated peripheral or plasma GABA levels may communicate with the brain to control food intake via hormonal or vagal afferent pathways, rather than by directly crossing the BBB and acting on the brain itself. Moreover, increased GABA levels induced by the drugs were not limited to the hypothalamus, the brain region central to food intake and appetite regulation, but were also observed in other brain regions, including the cortex and hippocampus. These findings suggest that the GABA-T inhibitory drugs (vigabatrin and EOS) can penetrate various brain regions, including the hypothalamus, cortex, and hippocampus, where they inhibit GABA-T activity, thereby increasing GABA levels in these areas. Taken together, it can be concluded that increased plasma or peripheral GABA, rather than increased brain GABA, is the key effector in suppressing food intake. However, in this study, brain tissues were collected in the afternoon (13:00-16:00) after a 6-h fast, which may not have captured the rapid changes in GABA levels or signals associated with the food-suppressive effect of the treatment. Future research should consider collecting brain tissues at different times, such as during refeeding, or employing other analytical techniques, such as brain immunohistochemical analysis, to better capture acute changes in brain signals related to food intake regulation. Although EOS has been reported to have slightly different properties compared to vigabatrin, such as lower BBB permeability (Storici et al., 2004), its ability to increase plasma and hypothalamic GABA levels is similar to that of vigabatrin (Figures 2A,B,D). Therefore, we hypothesized that EOS possibly exhibits characteristics similar to vigabatrin in suppressing food intake in our study.

To the best of our knowledge, this study is the first to demonstrate strong food intake-suppressive and weight-loss effects through a pharmaceutical-nutritional treatment comparable to the anti-obesity drug semaglutide. Given that GABA cannot cross the BBB and is extensively catabolized in the periphery, the role of dietary or peripheral GABA in the brain has not been extensively studied. However, recent research has highlighted GABA as a potential postbiotic mediator in the gut-brain axis, bringing renewed attention to the possible roles of dietary or peripheral GABA in brain functions (Kumrungsee, 2024; Braga et al., 2024 and the references therein). A recent study showed that oral administration of GABA (30 mg/kg daily) significantly prevented weight gain, reducing final body weight by 38% compared to control obese mice fed with a HF diet over 8 weeks (Jin et al., 2024). However, its anti-obesity effect was attributed to the suppression of adipogenesis and lipogenesis, with no reported impact on food intake suppression (Jin et al., 2024). Another study found that oral administration of GABA (200 mg/kg) reduced food intake by 15% during the first 30 min after refeeding (following 16 h of fasting), but this effect did not persist chronically, as food intake levels became comparable to those of control mice by the end of 24 h of refeeding (Nakamura et al., 2022). Interestingly, this brief food intake-suppressive effect (the first 30 min after refeeding) was abolished when GABA was administered 30 min before refeeding (Nakamura et al., 2022). Based on our present study, we hypothesize that during the first 30 min after oral GABA administration, GABA might be rapidly degraded by GABA-T in the liver, leading to a decrease in blood GABA levels that may fall below the threshold required to exert a food intake-suppressive effect. This observation supports our finding that increased blood GABA availability is a key factor in suppressing food intake. Although the study by Nakamura et al. (2022) did not specifically address the role of blood GABA levels in food intake suppression, it demonstrated that oral GABA administration suppresses food intake by sending peripheral signals through the vagus nerve to the brainstem (i.e., nodose ganglia, nucleus tractus solitarius, and area postrema). In earlier research on the anti-obesity effects of dietary GABA, most studies focused on its peripheral actions, such as anti-inflammatory and antioxidant effects, glucose metabolism, and beta-cell function (Hwang et al., 2019; Tian et al., 2011; Untereiner et al., 2019; Xie et al., 2014; Xie et al., 2015), without demonstrating its impact on food intake suppression and central control actions within the brain.

In addition to GABA, other amino acids have been reported to influence food intake regulation (Heeley and Blouet, 2016). Among these, leucine has been extensively studied. Research on central leucine infusion has consistently shown strong food intake suppression, largely through mechanisms such as the activation of hypothalamus-brainstem signaling pathways (Blouet et al., 2009; Pedroso et al., 2015). However, the effects of dietary or oral leucine administration have produced mixed results, often failing to suppress food intake (Pedroso et al., 2015). Similarly, neurotransmitter precursor amino acids, such as tyrosine, tryptophan, and histidine, have also been studied for their potential to regulate food intake. However, the evidence for their food intake-suppressive effects remains inconclusive (Kasaoka et al., 2004; Bassil et al., 2007; Fromentin et al., 2012). In contrast to amino acid supplementation, amino acid deficiency in diets also impacts food intake. For example, a diet deficient in lysine and tryptophan (essential amino acids) has been found to strongly suppress food intake in *Drosophila* larvae through the activation of GCN2, which in turn activates downstream pathways that reduce GABAergic inhibition in dopamine neurons (Bjordal et al., 2014). In rodents, GABA signaling in the anterior piriform cortex has been shown to play a role in regulating food intake in response to amino acid-deficient diets (Truong et al., 2002). These studies suggest that manipulating GABA signaling in the brain can modulate food intake. Despite these findings, amino acid-based interventions for food intake suppression and anti-obesity have not been well developed, likely due to the mixed and often controversial results mentioned above. While their central effects on food intake suppression are pronounced and well demonstrated, their peripheral effects remain highly controversial. It is possible that the availability of these amino acids in the blood or periphery may be a critical factor in their peripheral regulation of food intake. Based on our present findings, we hypothesize that increasing blood levels of amino acids, for example, by inhibiting their degradation pathways, could be a promising strategy for developing amino acid-based interventions for food intake suppression.

To advance the strategy of coadministration of peripheral GABA intake and GABA-T inhibitory drugs as a novel and effective anti-obesity intervention, further studies are essential to elucidate its peripheral and central mechanisms of action. On the peripheral level, it is necessary to examine the involvement of the vagus nerve, appetite-related gut hormones, and the effects on gastric emptying. On the central level, the roles of GABAergic, glutamatergic, and other neuropeptidergic neurons, as well as hypothalamus-brainstem circuits, need to be thoroughly investigated.

## Data Availability

The raw data supporting the conclusions of this article will be made available by the authors, without undue reservation.
